# COVID-19 healthcare cost and length of hospital stay in Turkey: retrospective analysis from the first peak of the pandemic

**DOI:** 10.1186/s13561-021-00338-8

**Published:** 2021-10-08

**Authors:** Ergun Oksuz, Simten Malhan, Mustafa Sait Gonen, Zekayi Kutlubay, Yilmaz Keskindemirci, Fehmi Tabak

**Affiliations:** 1 Department of Family Medicine, Baskent University, Faculty of Medicine, Ankara, Turkey; 2 Department of Health Care Management, Baskent University, Faculty of Health Sciences, Ankara, Turkey; 3 Istanbul University-Dean of Cerrahpasa Medical School, Istanbul, Turkey; 4 Istanbul University Hospitals General Director, Istanbul, Turkey; 5 Department of Infectious Disease, Istanbul University -Cerrahpasa Medical School, Istanbul, Turkey

**Keywords:** COVID-19, SARS-COV-2, Health care cost, Length of stay, Intensive care unit, Inpatient, Severity of the disease

## Abstract

**Background:**

During the COVID-19 pandemic, health care systems are under extreme pressure. This study analyzed health care resource use (HCRU) and costs in patients admitted to the hospital for COVID-19 and aimed to estimate the one-year direct medical cost of the disease in Turkey.

**Methods:**

This retrospective cohort study was conducted between March and July 2020 in a tertiary hospital (*n* = 1056) in Istanbul. Patient demographics, clinical and treatment characteristics at admission, comorbidities, disease severity, and costs from a payer perspective were evaluated using the microcosting method. The results include LOS, hospital costs, and univariate and generalized linear models to investigate influencing factors. The data were extrapolated to provide a country-level estimate.

**Results:**

The mean length of stay was 9.1 days (SD 6.9). The mean length of stay was 8.0 days (4.7) for patients hospitalized in wards versus 14.8 days (SD 12.0) for patients hospitalized in the ICU. In univariate analysis, several factors, including O_2_ therapy (+ 3.7 days), high CRP > 41.8 mg/L (+ 3.8 days), and elevated ferritin (+ 3.5), were found to be associated with a longer LOS (*p* < 0.05).

The direct annual medical cost of COVID-19 was estimated at PPP$ 2.1 billion. The COVID-19 pandemic resulted in a direct medical burden that corresponds to 2.0% of the government health expenditures and 0.8 per thousand of Turkey’s gross domestic product (GDP).

**Conclusions:**

Estimating the impact of this pandemic in terms of HCRU and costs to the health care system can help design strategies to manage the pandemic.

**Supplementary Information:**

The online version contains supplementary material available at 10.1186/s13561-021-00338-8.

## Background

By the end of 2020, COVID-19 affected the entire world, with over 83 million cases and approximately 2 million deaths [1]. Many countries have closed their borders, stopped all flights, and had to reduce their affairs with other countries. These measures caused significant shrinkage in economies worldwide, with businesses closing, increasing unemployment, rising inflation, and the interruption of production and shipping [2]. On an individual level, the global COVID-19 pandemic caused significant stress worldwide, as people were required to quarantine in their homes for long periods of time [3]. During the first peak of the pandemic, serious health care challenges were experienced around the world. There was no specific treatment for SARS-COV-2, and extraordinary efforts were made to find an effective treatment to reduce morbidity and mortality [4].

World leaders were already warning of the sustainability of health care systems before the current pandemic hit [5, 6]. The rapid and global spread of the pandemic has exacerbated existing problems and created new issues that will challenge decision-makers and negatively impact the health of the populace [7–9]. To mitigate the impact of this and future pandemics, decision-makers need to understand how their health systems were impacted. Of particular importance is understanding the health care resource use (HRCU) (e.g., length of hospital stay) and subsequent costs of the pandemic [4]. Decision-makers can use this knowledge to plan resources and budget allocation for future health crises.

To date, few studies have investigated the extent of HRCU and direct health care costs. This study utilizes real-world data to calculate the HRCU and costs using a microcosting approach in Turkey, one of the countries with the highest number of SARS-CoV-2 cases (2.5 million infections and approximately 27,000 deaths as of February 2021) in the world [10].

## Methods

A single-center, retrospective study was conducted in a tertiary hospital (Istanbul Cerrahpasa University Hospital) in İstanbul, Turkey. The data were obtained via the Hospital Data Management System (HDMS; including financial records, accounting records, and clinical patient files) and consisted of a total of 2972 hospitalizations of 2284 patients with SARS-CoV-2 in the given period.

### Patient inclusion/exclusion

**Fig. 1:**
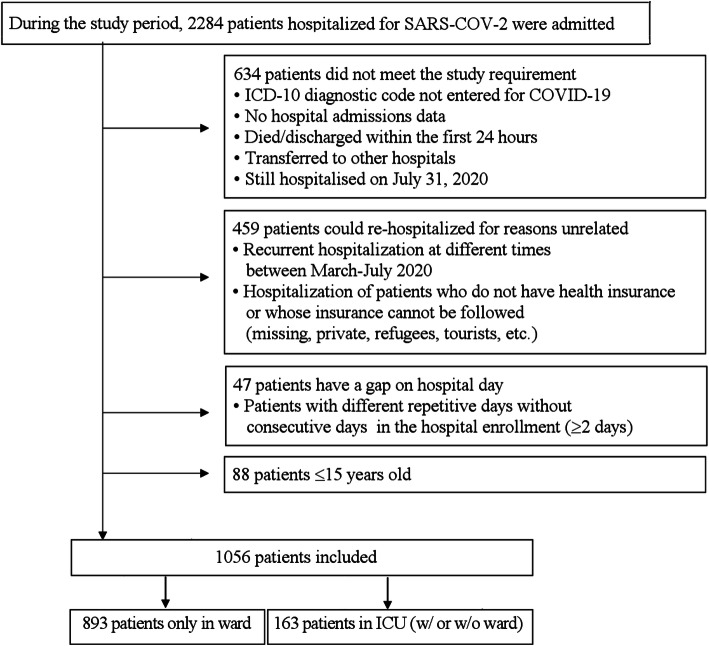
Flow chart of the study cohort

The study sample included all adult patients (≥16 years of age) admitted with SARS-CoV-2 infection confirmed by real-time reverse transcription-polymerase chain reaction (PCR) and/or chest computed tomography (CT) between March 11 and July 31, 2020 (the first 20 weeks of the pandemic in Turkey). Patients were excluded if they did not have an ICD 10 diagnosis or admission code recorded in the HDMS, were still hospitalized as of July 31, 2020, had evidence of recurrent hospitalizations, had no health insurance or were transferred to different departments (see Fig. 1 for the cohort flow chart).

### HRCU and costing

A bottom-up and microcosting method was used to estimate the HRCU and associated costs from the Turkish payer perspective (The Republic of Turkey, Ministry of Family, Labor and Social Services Social Security Institution - SSI) [11]. Unit costs were retrieved from the Health Implementation Notification (HIN) of the SSI; the type and frequency of services used by patients as well as their utilization percentage were retrieved from the HDMS. All costs were calculated in Turkish Lira (TRY), and to assist comparisons, all costs were converted into 2019 Purchasing Power Parities (PPP$) using the Organization for Economic Co-operation and Development (OECD) rates (1 TRY = PPP$ 1.841) [12].

Within the SSI reimbursement system, the “payment based on procedure” method is used for certain health care services. In this payment scheme, health care costs are billed in packages. This system is similar to the Health Care Common Procedure Coding System (HCPCS) or Current Procedural Terminology (CPT Codes). In cases listed under this payment, the bed fee, examinations and consultations, operations and interventions, certain medications, consumables, laboratory, pathology, radiology examinations, and anesthesia procedures are not invoiced separately. The SSI in Turkey has made payments to the hospitals based on the daily procedures performed, under the name “pandemic care payment”, initially for patients who were PCR positive and were hospitalized in the ICU (as of April 1, 2020) (for COVID-19 ward hospitalization PPP$ 370.4/day, and for hospitalization in the ICU, based on the ICU levels, PPP 119.5/day, PPP 253.9/day, and PPP 478.3/day). The health care resource utilization components that are included in services received in return for such payments are not standard for all patients. Their distribution has not been calculated, and they are among the other package payments. They are referred to as procedure package payments in the analysis.

### Statistical methods

Descriptive statistics (mean and standard deviation (SD)) were used to evaluate the outpatient examinations, emergency room admissions, hospital admissions, ICU admissions, laboratory and imaging tests, medical procedures, and package procedures based on diagnosis, drugs, and medical supplies used and stratified by gender. Total hospital costs were determined for subgroups based on age, sex, complaints at admission, physical examination and vital signs, number of comorbidities, hematologic, coagulation, inflammatory and biochemical biomarkers, PCR tests, and respiratory status (chest CT, SpO_2_, O_2_ therapy).

Continuous variables are expressed as the mean (SD) and median (interquartile range - IQR); categorical variables are expressed as numbers, percentages, and 95% confidence intervals. Pearson, Yates, Fisher’s exact and likelihood chi-square tests were used to compare the groups’ characteristics. A descriptive analysis was conducted to compare the costs that were categorized into different groups. Comparisons (Student’s t-test or the Mann–Whitney test) were made for variables with a normal distribution. T-tests and Welch’s ANOVA test were used to compare the means. Cohen’s d calculation was performed to determine the effect size. The existence of heteroscedasticity was investigated, as there may be estimators with a heteroscedasticity bias that can be found specifically due to the specificity of the estimation of cost functions (Supplementary Fig. 1). Using the resulting weighting, the determinants of health care costs were analyzed using a generalized linear model (GLM) with the gamma distribution and log link function [13]. Adjusted odds ratios (ORs) and 95% confidence intervals (CIs) were calculated according to this model.

Demographic, clinical, and treatment cost data were collected from the electronic patient record system, and Microsoft Excel 2016 (Microsoft Corp, Redmond, WA) software was used for raw data entry. During the analysis for December 2020 and January 2021, there were no official figures for the number of patients hospitalized in Turkey. The linear least squares regression (LSM) method was used to determine the total number of hospitalized patients during these two months. Statistical calculations were performed using SPSS 24.0 (IBM Corp, Armonk, NY) software. *P* values of less than 0.05 were considered statistically significant.

## Results

After applying the inclusion/exclusion criteria, a total of 1056 patients were included for analysis (Fig. 1).

### Patient baseline characteristics

Of 1056 patients, 55% were men and 45% were women. The mean age was 56.6 years (16–98 years) (Table 1). A total of 84.6% were treated in wards only (*n* = 893), while 15.4% were hospitalized in the ICU (*n* = 163). ICU hospitalization rates were significantly higher among men (18.0%) than women (12.2%). Patients presented with twenty-nine different symptoms at admission. Sixteen symptoms were reported in more than 5 patients. The most common symptoms reported were cough (36.7%), fever (32.1%), dyspnea (30.5%) and malaise (18.6%). Fever was most common in men, while myalgia, headache, and chest/back pain were more common in women (*p* < 0.05). The distribution of the groups based on COVID-19 severity was presymptomatic 5.9%, mild 17.0%, moderate 41.2% and severe 36.0% (Supplementary Table 1).

**Table 1:** Baseline patient demographics, symptoms, findings, and fatality by COVID-19 severity

Demographics				
No. of cases	1056			
**Sex**				
Male	582			
Female	474			
Ratio M:F	1.2:1.0			
Age of patients - mean years	56.6			
Range (years)	16–98			
**COVID-19 Severity**	**Pre-symptomatic /Mild** **n (%)**	**Moderate** **n (%)**	**Severe** **n (%)**	***p*-value**
**Sex**				
Male	124 (51.5)	238 **(**54.7)	220 **(**57.9)	0.283
Female	117 **(**48.5)	197 **(**45.3)	160 **(**42.1)	
Total	241 (22.8)	435 (41.2)	380 (36.0)	
**Clinical features**				
Cough	72 (29.9)	194 (44.6)	122 (32.1)	0.000
Fever	72 (29.9)	141 (32.4)	126 (33.2)	0.683
Dyspnea	0 (0.0)	168 (38.6)	154 (40.5)	0.000
Malaise	37 (15.4)	81 (18.6)	78 (20.5)	0.271
Myalgia	25 (10.4)	35 (8.0)	14 (3.7)	0.003
Nausea or vomiting	11 (4.6)	35 (8.0)	19 (5.0)	0.099
Diarrhea	15 (6.2)	25 (5.7)	25 (6.6)	0.884
Headache	15 (6.2)	24 (5.5)	18 (4.7)	0.719
Sputum	6 (2.5)	16 (3.7)	16 (4.2)	0.529
Sore throat	13 (5.4)	15 (3.4)	9 (2.4)	0.135
Chills or rigors	7 (2.9)	15 (3.4)	12 (3.2)	0.926
Chest pain or back pain	5 (2.1)	17 (3.9)	8 (2.1)	0.217
Anorexia	4 (1.7)	11 (2.5)	10 (2.6)	0.710
Abdominal pain	5 (2.1)	5 (1.1)	5 (1.3)	0.608
Loss of smell or taste	3 (1.2)	4 (0.9)	5 (1.3)	0.854
Rhinorrhoea/Stuffiness	3 (1.2)	3 (0.7)	1 (0.3)	0.339
Others	10 (4.1)	15 (3.4)	26 (6.8)	0.067
**Hematologic biomarkers**				
Lymphopenia	50 (20.7)	95 (21.8)	145 (38.2)	0.000
Thrombocytopenia	49 (20.3)	74 (17.0)	109 (28.7)	0.000
Neutrophilia	31 (12.9)	62 (14.3)	84 (22.1)	0.002
**Coagulation biomarkers**				
Elevated D-dimer	126 (52.5)	268 (61.6)	269 (71.0)	0.000
Elevated fibrinogen	187 (82.4)	331 (84.9)	319 (87.2)	0.277
**Inflammatory biomarkers**				
Elevated C-reactive protein				
*3.1–41.8 mg/L*	110 (45.6)	215 (49.4)	135 (35.5)	0.000
*> 41.8 mg/L*	79 (32.8)	176 (40.5)	219 (57.6)	
Elevated serum ferritin	45 (20.1)	88 (21.4)	142 (38.3)	0.000
**Biochemical biomarkers**				
Elevated LDH	51 (21.7)	143 (33.2)	184 (48.5)	0.000
Elevated liver enzymes	51 (21.2)	103 (23.7)	130 (34.2)	0.000
Elevated serum blood urea	201 (83.4)	348 (80.4)	331 (87.3)	0.028
Elevated serum blood creatinine	34 (14.1)	80 (18.5)	90 (23.7)	0.010
**Chest CT results**				
Positive	0 (0.0)	392 (95.6)	214 (88.4)	0.000
**PCR results**				
Positive	132 (55.5)	203 (48.3)	202 (55.0)	0.095
**Comorbidities**				
Comorbidity presence (any)	100 (41.5)	201 (46.2)	192 (50.5)	0.086
Hypertension	44 (18.3)	106 (24.4)	102 (26.8)	0.048
Diabetes	25 (10.4)	59 (13.6)	60 (15.8)	0.159
Asthma/COPD	13 (5.4)	44 (10.1)	39 (10.3)	0.075
Ischemic heart disease	15 (6.2)	29 (6.7)	42 (11.1)	0.034
Cancer	15 (6.2)	24 (5.5)	41 (10.8)	0.012
Chronic renal disease	8 (3.3)	23 (5.3)	16 (4.2)	0.474
Heart failure	3 (1.2)	21 (4.8)	20 (5.3)	0.034
**Inpatient settings**				
Ward only	227 (94.2)	388 (89.2)	278 (73.2)	0.000
Ward &ICU	14 (5.8)	47 (10.8)	102 (26.8)	
Fatality (in hospital)	6 (2.5)	26 (6.0)	68 (17.9)	0.000

CT: Computed tomography; ICU: Intensive care unit; COPD: Chronic obstructive pulmonary disease; PCR: Polymerase chain reaction

Among the hematologic biomarkers, lymphopenia was detected in 27.5%, thrombocytopenia in 22.0%, and neutrophilia in 16.9%. Lymphopenia and thrombocytopenia were more common in men (p < 0.05). Among the coagulation biomarkers, 62.9% of the patients had elevated D-dimer, and 85.1% had elevated fibrinogen. Elevated fibrinogen was more common in men (*p* < 0.05). In the inflammatory biomarker group, elevated CRP > 3.0 mg/L was present in 88.5% of the patients, > 41.8 mg/L in 44.9% of the patients, and elevated serum ferritin was present in 27.3% of the patients. Elevated inflammatory markers were observed more commonly in men (p < 0.05). Elevated LDH was detected in 36.2% of the patients, elevated liver enzymes (AST/ALT) in 26.9%, elevated serum blood urea in 83.6% and elevated serum blood creatinine in 19.4% of the patients (Supplementary Table 1).

Lung involvement was present in 79.5% of CT scans, and 37.0% of the patients had SpO_2_ < 94%. PCR testing was conducted twice in all patients, and the results were positive in 52.4%. A total of 46.7% of the patients had at least one comorbidity, and it was more common in women (p < 0.05). Comorbidities included hypertension (23.9%), diabetes mellitus (13.6%), asthma/chronic obstructive pulmonary disease (COPD) (9.1%), ischemic heart disease (IHD) (8.1%), cancer (7.6%), chronic renal disease (CRD) (4.5%) and heart failure (HF) (4.2%) (Supplementary Table 1).

The incidence of severe COVID-19 was higher in men than in women, but the difference was not significant (42.1% vs. 57.9%; *p* = 0.283). The most common symptom was dyspnea (40.5%) in severe COVID-19 patients, cough in the moderate group (44.6%), and cough (29.9%) and fever (29.9%) in the presymptomatic/mild group. Cough was observed at a higher rate in the moderate and severe groups (*p* = 0.000). The percentage of dyspnea was 38.6% in the moderate COVID-19 group and 40.5% in the severe group (p = 0.000). Myalgia was observed more frequently in the presymptomatic/mild COVID-19 group than in the other groups (10.4% versus 3.7% in severe and 8.0% in moderate; *p* = 0.003). The incidence of lymphopenia and neutrophilia increased with the severity of COVID-19. Lymphopenia was observed in 38.2% (*n* = 145) of severe COVID-19 patients, and neutrophilia was observed in 22.1% (*n* = 84). While the incidence of elevated D-dimer was 52.5% in the presymptomatic/mild group, it increased up to 71.0% in the severe group (*p* = 0.000). The incidence of elevated inflammatory biomarkers increased as the severity of the disease increased. CRP levels of > 41.8 mg/L were observed in 57.6% of severe COVID-19 patients, and elevated ferritin was observed in 38.3% of the same group. Elevations of all biochemical biomarkers was more frequent in the severe COVID-19 group. Although comorbidities in the severe COVID-19 group were slightly more common than those in the other groups, there was no significant difference. Hypertension, IHD, cancer, and HF were more common in the severe COVID-19 group than in the other groups (*p* < 0.05). In the presymptomatic/mild group, 5.8% of the patients required treatment in the ICU. This rate was 10.8% in the moderate group and 26.8% in the severe group (*p* = 0.000). The in-hospital fatality rate was 17.9% in the severe group, 6.0% in the moderate group, and 2.5% in the mild COVID-19 group (p = 0.000) (Table 1).

### Resource use and costs

The mean LOS was 9.1 ± 6.9 days, and the median was 7.0 days (IQR 6.0) (1.0–93.0 days). The mean cost of COVID-19 episodes per patient was PPP $5,557.9 ± 7,473.4, and the median was PPP $3,585.9 (IQR 3,754.5) (PPP $45.6–96,130.1). The largest cost item was procedural packages at 64.4%, followed by drugs at 9.9%, laboratory tests at 9.6%, bed at 7.4%, interventions at 4.6%, medical supplies at 3.3%, imaging tests at 0.7%, and physician costs at 0.2% (Table 2).

**Table 2:** Length of Hospital Stay, Mean Cost of Episode Per Patient and Cost Components -PPP$

	Ward (***n*** = 893)			ICU (***n*** = 163)			Total (***n*** = 1056)		
	Mean (SD)	Median (IQR)	Min-Max	Mean (SD)	Median (IQR)	Min-Max	Mean (SD)	Median	Min-Max
Length of stay (days)	8.0 (4.7)	7.0 (5.0)	1.0–54.0	14.8 (12.0)	13.0 (12.0)	1.0–93.0	9.1 (6.9)	7.0 (6.0)	1.0–93.0
Costs (PPP$)									
Physician costs	10.3 (18.9)	3.7 (11.0)	0.0–332.1	17.5 (24.3)	11.0 (18.3)	0.0–198.0	11.4 (20.0)	3.7 (14.7)	0.0–332.1
Laboratory tests	543.7 (353.0)	480.9 (355.8)	0.0–3,739.5	465.4 (458.7)	321.2 (478.8)	0.0–3,023.5	531.6 (372.1)	465.3 (389.9)	0.0–3,739.5
Imaging tests	37.0 (101.1)	12.4 (20.7)	0.0–.1,249.0	34.0 (79.2)	5.5 (42.5)	0.0–792.2	36.7 (98.0)	12.4 (22.7)	0.0–1,249.0
Bed	427.9 (273.7)	361.7 (285.6)	24.4–2,488.9	314.8 (349.3)	173.6 (333.9)	0.0–2,347.3	410.4 (289.4)	337.2 (297.8)	0.0–2,488.9
Interventions	249.4 (249.7)	204.3 (158.2)	0.0–3,887,0	277.7 (583.5)	132.9 (290.6)	0.0–5,741.8	253.7 (324.2)	192.6 (177.9)	0.0–5,741.8
Drugs	312.9 (660.5)	95.1 (247.1)	0.0–9,421.7	1,843.1 (2,442.7)	958.3 (2,115.9)	0.0–18,497.7	549.1 (1,261.4)	115.1 (412.9)	0.0–18,497.7
Medical supplies	19.2 (132.0)	6.1 (10.7)	0.0–3,158.3	1082.9 (2,630.9)	231.4 (499.2)	0.0–17,100.3	183.4 (1,107.0)	7.1 (16.1)	0.0–17,100.3
Procedural packages	2,345.7 (1,856.8)	2,222.5 (2,222.5)	0.0–19,631.8	10,352.7 (11,281.7)	7,256.8 (11,215.1)	0.0–74,256.5	3,581.6 (5,553.0)	2,222.5 (2,222.5)	0.0–74,256.5
Total costs	3,946.2 (2,918.9)	3,294.2 (2,996.7)	45.6–30,201.4	14,388.1 (14,968.0)	9,956.2 (13,341.2)	80.5–96,130.1	5,557.9 (7,473.4)	3,585.9 (3,754.5)	45.6–96,130.1

SD: Standard deviation; IQR: Interquartile range; Min: minimum; Max: Maximum; ICU: Intensive care unit; PPP: Purchasing power parity

### General Ward admissions

The mean length of stay in the general wards was 8.0 ± 4.7 days, with a median of 7 days (IQR 5.0) (Table 2). When we analyzed the cost of 893 COVID-19 patients who received health care services only in hospital wards, we identified the highest cost item as procedural packages at 59.4%; followed by laboratory tests at 13.8%, bed at 10.8%, drugs at 7.9%, interventions at 6.3%, imaging tests at 0.9%, medical supplies at 0.5% and physician costs at 0.3%. The mean costs of the items were as follows: physician costs PPP$ 10.3 ± 18.9, laboratory tests PPP$ 543.7 ± 353.0, imaging tests PPP$ 37.2 ± 101.1, bed costs PPP$ 427.9 ± 273.7, interventions PPP$ 249.4 ± 249.7, drugs PPP$ 312.9 ± 660.5, medical supplies PPP$ 19.2 ± 132.0, and procedural packages PPP$ 2,345.7 ± 1,856.8. The total cost per patient was PPP$ 3,946.2 ± 2,918.9.

### ICU admissions

One hundred and sixty-three COVID-19 patients were admitted to the ICU. The mean length of stay in the ICU was 14.8 ± 12.0, and the median was 13.0 (IQR 12.0) (Table 2). Fifty-eight (35.0%) patients were directly admitted to the ICU at presentation, and 105 (65.0%) were transferred to the ICU from the general ward. The mean length of stay in the general ward was 5.9 ± 4.6 days and 17.3 ± 12.4 days in the ICU for these patients. The mean costs were as follows: physician costs PPP$ 17.5 ± 24.3, laboratory tests PPP$ 465.4 ± 458.7, imaging tests PPP$ 34.0 ± 79.2, bed costs PPP$ 314.8 ± 349.3, interventions PPP$ 277.7 ± 583.5, drug costs PPP$ 1,843.1 ± 2,442.7, medical supplies PPP$ 1,082.9 ± 2,630.9, and procedural packages PPP$ 10,352.7 ± 11,281.7. The total cost was PPP$ 14,388.1 ± 14,968.0. The largest share of the ICU costs was procedural packages at 72.0%, followed by drugs at 12.8%, medical supplies at 7.5%, laboratory tests at 3.2%, beds at 2.2%, interventions at 1.9%, imaging tests at 0.2%, and physician costs at 0.1%.

### Resource use and costs based on COVID-19 severity

In the presymptomatic/mild COVID-19 group (*n* = 241), the mean LOS was 7.3 ± 4.5 days with a median of 7.0 days (IQR 4.0 days). There were marginal differences for this group between the general ward and ICU stays. The mean LOS for moderate COVID-19 patients (*n* = 435) was 8.6 ± 6.0 days, and the median LOS was 7.0 days (IQR 5.0 days). There was a mean difference of approximately 2 days between the general ward and ICU stays in the moderate group. For patients with severe COVID-19 (*n* = 380), the mean LOS was 10.8 ± 8.4 days, the median was 8.0 days (IQR 7.0 days), the mean LOS in the general ward was 8.3 ± 5.3 days and in the ICU 11.9 ± 12.6 days (Welch’s F (2,663.2) = 21.60, *p* = 0.000, est. ω2 = 0.038) (Table 3).

**Table 3:** Hospital Costs and Length of Stay by Disease Severity and Post Hoc Results

COVID-19 Severity	Pre-symptomatic/Mild	Moderate	Severe
Length of stay (days)*			
Ward	7.1 (4.4)	7.8 (4.5)	8.3 (5.3)
ICU	7.6 (5.0)	10.1 (11.0)	11.9 (12.6)
Total	7.3 (4.5)	8.6 (6.0)	10.8 (8.4)
Mean Differences (Cohen d’s)			
vs. *pre-syptomatic/mild*	–	1.2 (0.2) ***	3.4 (0.5) #
vs.*moderate*	–	–	2.2 (0.3) #
Cost (PPP$)**			
Physician costs	8.9 (26.6)	10.9 (16.5)	13.5 (18.5)
Laboratory tests	487.3 (371.7)	526.3 (353.9)	565.7 (389.9)
Imaging tests	27.3 (64.4)	34.2 (95.4)	45.7 (116.4)
Bed	345.7 (263.4)	438.4 (288.2)	419.4 (300.8)
Interventions	228.5 (349.3)	261.7 (362.5)	260.6 (253.7)
Drugs	370.5 (927.7)	368.3 (1087.7)	869.3 (1538.9)
Medical supplies	35.7 (228.7)	116.8 (924.5)	353.4 (1533.4)
Procedural packages	2,290.9 (2141.4)	3,127.4 (5025.2)	4,920.1 (7135.9)
Total	3,794.7 (3275.5)	4,883.9 (6991.2)	7,447.7 (9307.5)
Mean Differences (Cohen d’s)			
vs. *pre-syptomatic/mild*	–	1,089.2 (0.2) ***	3,626.6 (0.5) #
vs.*moderate*	–	–	2,556.5 (0.3) #

ICU: Intensive care unit; PPP: Purchasing power parity

* ANOVA: Welch’s F (2, 663.2) = 21.60, *p* = 0.000, est. ω2 = 0.038

**ANOVA: Welch’s F (2, 682.7) = 25.27, *p* = 0.000, est. ω2 = 0.044

*** *p* < 0.05, # *p* = 0.000

**Fig. 2:**
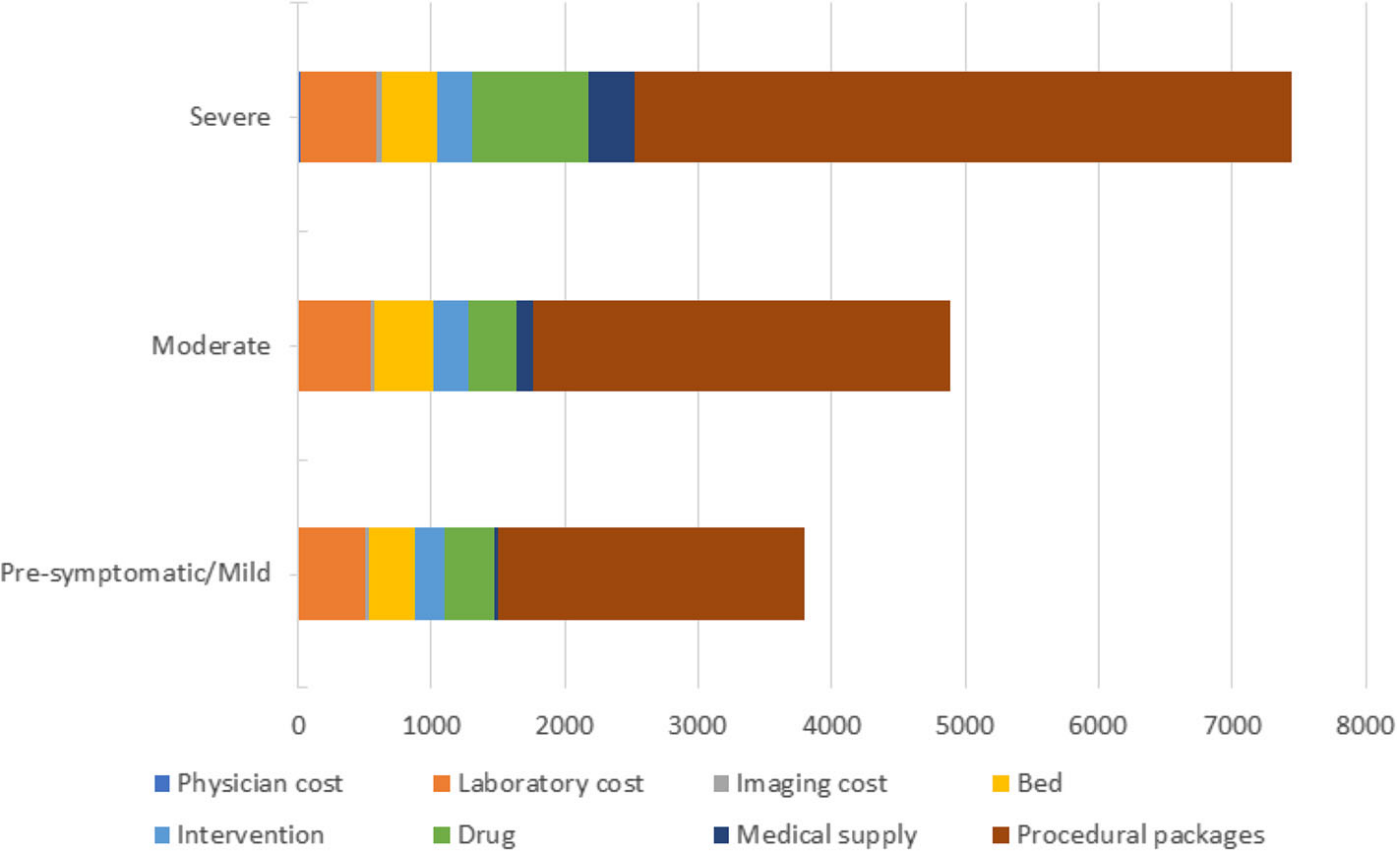
Distribution of Cost Components by Disease Severity

Based on the severity of the disease, the costs of laboratory tests, beds, and interventions were higher in presymptomatic/mild COVID-19 patients than in the other groups. In comparison, drugs and medical supply costs were higher in severe COVID-19 patients. Procedural package costs (including the service package cost included in the reimbursement during the pandemic) increased from the presymptomatic to the severe group. They accounted for 60, 64, and 66% of the total cost, respectively. When we excluded the procedural package costs component, the presymptomatic/mild and moderate COVID-19 groups’ highest cost components were laboratory tests. In contrast, the highest cost component for the severe COVID-19 groups was drug costs (Welch’s F (2, 682.7) = 25.27, *p* = 0.000, est. ω2 = 0.044) (Table 3) (Fig. 2).

In the post hoc analysis, severe COVID-19 led to a longer length of stay; compared to presymptomatic/mild disease, it had a 3.4-day mean difference in LOS with a medium effect size (Cohen’s d = 0.5, *p* = 0.000), and compared to moderate disease, a 2.2-day mean difference in LOS with a small effect size (Cohen’s d = 0.3, p = 0.000). Moderate COVID-19 resulted in a longer LOS with a 1.2-day mean difference and a small effect size (Cohen’s d = 0.2, *p* < 0.05) compared to presymptomatic/mild COVID-19 (Table 3).

Severe COVID-19 resulted in higher hospital costs; compared to presymptomatic/mild disease, it showed a PPP$ 3,626.6 mean difference in hospital costs with a medium effect size (Cohen’s d = 0.5, *p* = 0.000), and compared to moderate disease, it showed a PPP$ 2,556.5 mean difference in hospital costs with a small effect size (Cohen’s d = 0.3). Moderate COVID-19 resulted in higher hospital costs with a PPP$ 1,089.2 mean difference and a small effect size (Cohen’s d = 0.2, p < 0.05) compared to presymptomatic/mild COVID-19 (Table 3).

Factors causing a longer LOS and higher hospital costs were investigated in the univariate analysis. According to this analysis, patients with a fatality outcome required 5.1 days (95% CI 3.8–6.5, *p* = 0.000) longer hospitalization. The LOS was significantly (p = 0.000) longer in the patients who had CRP > 41.8 mg/L (3.8 days), received O_2_ therapy (3.7 days), had elevated ferritin levels (3.5 days), had lymphopenia (2.6 days) and patients with SpO_2_ < 94% (2.5 days). Although patients with comorbidities such as hypertension, asthma/COPD, IHD, cancer, CRD, and HF had longer LOSs than those without comorbidities, the mean differences were not statistically significant.

The cost per patient in patients with neutropenia, lymphopenia, elevated ferritin, CRP > 41.8 mg/L and those patients who died was significantly (p = 0.000) higher than that in patients who did not have these outcomes (Supplementary Table S2). The cost differences were not significant between patients with or without thrombocytopenia, diabetes, asthma/COPD, or IHD (Supplementary Table S2).

Costs were significantly (4.0 times) higher (95% CI 2.0–7.9; p = 0.000) in patients with positive PCR test results than in patients with negative PCR test results. The costs for patients who received O_2_ therapy were 2.0 times higher (95% CI 1.3–3.2; *p* = 0.002) than those who did not receive O_2_ therapy. Although the mean costs in the severe COVID-19 group were higher than those in the presymptomatic and mild COVID-19 groups (OR 1.1, 95% CI 0.4–2.6, *p* = 0.937), the difference was not significant. Age > 65 was independently associated with higher costs. The costs for female patients over 65 years of age were 5.8 times higher (95% CI 1.6–21.5; *p* = 0.008), while those of male patients over 65 years of age were 4.6 times higher (95% CI 1.1–18.9; *p* = 0.035). Direct medical costs were also significantly higher for patients with elevated LDH and neutrophilia than for those without these problems at baseline. According to the multivariate model, chronic comorbidities could result in higher hospital costs, and these results were significant for chronic renal disease, hypertension, and ischemic heart disease (Supplementary Table S3). Interactions between factors had no significant effects other than gender and age (Supplementary Table S4).

For the 62 outpatients who were followed up in the same period, the mean cost of outpatient COVID-19 episodes per patient was PPP$ 220.0. Overall, the largest cost items were laboratory tests (62.3%), drugs (16.7%), imaging tests (14.2%), and physician costs (6.8%).

### National burden

Using the available national statistics [14], the hospitalization rate was identified. The estimated number of inpatients was 253,118 as of January 31, 2021; the estimated mean cost per patient was PPP$ 5,557.9, and the estimated total inpatient cost was PPP$ 1,406,815,838.0 for this period. The total number of outpatients was 2,214,345, the mean cost per patient was PPP$ 220.0, and the estimated total outpatient cost was PPP$ 489,303,519.0. The estimated total cost of outpatients and inpatients for this period (11 months) was PPP$ 1.9 billion and PPP$ 2.1 billion annually.

Turkey had an estimated population of 84.3 million in 2020. Turkey’s estimated gross domestic product (GDP) in 2020 was PPP$ 2.5 trillion [15]. The estimated government health expenditure was PPP$ 104 billion, and the payer’s (SSI) health expenditure was a share of 51.7% (approximately PPP$ 54 billion) [16]. The cost of hospitalized COVID-19 patients accounted for 1.3% of the government annual health expenditures and 2.6% of SSI expenditures. The cost of outpatients accounted for 0.5% of government health expenditures and 0.9% of SSI health expenditures. The total cost is thought to constitute 2.0% of government health expenditures and 3.8% of SSI health expenditures. The average annual per capita government spending in Turkey is estimated at PPP$ 672.3. This suggests that the spending is 8.3 times higher for inpatients. During one year of the pandemic, the annual direct medical costs of COVID-19 caused a medical cost burden that corresponded to 0.8 per thousand of Turkey’s GDP (Table 4).

**Table 4:** Indicators for Turkey and COVID-19 Direct Medical Costs Burden - 2020

Population - Turkey	84,302,535
Gross domestic product (PPP $)	2.525 Tn
Total Health care Expenditure (Government) (PPP $)	104.3 Bn
*Social Security Institution (PPP$)*	53.9 Bn
Average per capita annual health care expenditure (PPP $)	672.3
**COVID-19 cases (n)**	
Outpatient	2,224,345
Inpatient	253,118
Total	2,477,463
**Direct Medical Costs - per patient (PPP $)**	
Outpatient	220.0
Inpatient	5,557.9
**Direct Medical Costs – Total (PPP $)**	
Outpatient	489,303,519
Inpatient	1,406,815,838
Total	1,896,119,357
Annual	2,068,493,844
**Burden (%)**	
The ratio in SSI health care expenditures	2.0%
The ratio in government health care expenditures	3.8%
The ratio in Turkey’s GDP	0,08%

PPP: Purchasing power parity;GDP: Gross domestic product

## Discussion

This study provides an estimate of the impact of the COVID-19 pandemic on the use of health care resources. Health care resource utilization costs were estimated based on cost components and patient characteristics. This study demonstrated that disease severity and certain clinical and demographic characteristics (e.g., age, PCR positivity, sex, receiving O_2_ therapy) had a significant impact on both LOS and direct medical costs.

While it is difficult to compare across the literature due to differences in methodology, population, health care costs, etc., studies from China [17], Switzerland [4], Saudi Arabia [18], Turkey [19] [20] and the US [21] all found that the burden on the health care system in terms of resource use and costs was substantial. In an early study conducted with 70 patients in China, the cost of an episode per patient was identified as USD 6827, and the highest cost item was drugs at 45.1% [17]. Another study from the US estimated the potential health care costs associated with percent infected population costs to be between $163.4 billion and $654.0 billion [21]. This disease is reported to have a catastrophic impact on health care expenditures and the world’s economies [22, 23]. Apart from the burden of health costs, the other burdens it caused were also quite high. For example, in the UK, school closures alone caused an economic burden of £166 bn (7.3% of GDP) and business closures £502 bn (21.9% of GDP) and resulted in a total burden of £668 bn (29.2% of GDP) on the British economy [24].

When we look at the pandemics the world has suffered thus far, H1N1 influenza (1918) caused the loss of 50 million people, 3% of Australia’s GDP, 15% of Canada’s GDP, 17% of the UK’s GDP, and 11% of the USA’s GDP. SARS (2003) caused 774 deaths and a total global economic burden of USD 52.2 billion. Ebola (2013) caused 10,600 deaths and GDP losses in Guinea, Sri Lanka, and Liberia of USD 2.8 billion. Zika (2015–2016) caused 20 deaths and a loss of $3.5 billion in Latin America and the Caribbean region [25]. Researchers have shown that health expenditure is a key factor that can be used to improve economic growth and development [26]. With the COVID-19 pandemic, the world saw the importance of health investments for the economy of countries. The pandemic has had devastating effects not only on health but also in many areas. These devastating effects far exceeded the health costs. The COVID-19 outbreak also caused significant economic damage due to restrictions imposed to prevent the virus from spreading. The International Monetary Fund estimates the cost of COVID-19 at three trillion Euros for the European Union [27]. The pandemic has caused relief for the ecosystem due to the lower than expected pollutant emissions due to limited sectoral activities (including transport, energy, finance sectors) and industry. Even so, the economic impact of the COVID-19 pandemic has jeopardized the economic outlook for all countries around the world. COVID-19 has created varying degrees of pandemic uncertainty around the world, so most governments have taken drastic measures or implemented partial restrictions, taking into account economic damage [28].

In Turkey, government policies to reduce the number of COVID-19 infections continue to be implemented. Schools were closed as of April 2020, and online education started, the working hours of shopping centers were shortened, lockdowns were imposed on the weekends, all restaurants and entertainment venues were closed, time restrictions were imposed on people older than 65 and younger than 18 when leaving their residence, some businesses benefited from the short-term work allowance scheme of the government, working hours were limited, home-office working was adopted, and flights to many countries were grounded. Despite measures to counter the pandemic in Turkey, the fluctuating number of cases within a year caused a significant burden on the local economy and an excess demand for health care institutions and health care professionals. Additionally, there is evidence that out-of-pocket expenditures were especially substantial in the lower socioeconomic group and increased the mortality rate [23].

### Limitations

This study was based on 11 months of real-world data from one tertiary hospital in Turkey. Only direct medical costs in the hospital setting were considered. It was not possible to use real-world data to investigate community-based care costs, PPE equipment costs, transport costs, surveillance efforts or other impacts on the health care system. This study also does not consider indirect costs or out-of-pocket costs to patients. In addition, only patients with insurance were considered in this analysis.

The analysis was performed using the highly sensitive microcosting method. This study is an estimation of the burden of COVID-19 across the country based on data obtained from a tertiary health care facility. Costs associated with out-of-hospital resources used outside the hospital, such as additional physician visits and patient transfers to the hospital or transportation to the hospital, were excluded. In the first months of the pandemic, there was also the cost of safely transporting PCR test samples to the central laboratories (PCR tests were initially performed only at the designated Public Health Laboratories in Istanbul, but one month later, serological tests were being analyzed at four centers). Among the pandemic control strategies, the measures implemented for the safety of health care professionals were quite extensive. Personal protective gowns, gloves, masks such as N95, FFP2, FFP, and face and eye shields had to be used during contact with patients. Efforts such as repurposing existing areas or creating special sections isolated from other parts of the hospital to provide separate testing environments also incurred additional costs. In addition, performing COVID-19 diagnostic tests on every patient before all surgical and interventional procedures, even without the presence of COVID-19 symptoms, caused additional costs of care for other diseases. We were unable to account for all of these costs. Furthermore, the costs of the long-term complications associated with COVID-19 or the side effects of the treatments could not be accounted for due to uncertainty.

If we consider the surveillance cost of the index patient’s family and other contacts, which should be included in the overall medical costs, the numbers would be far above our medical cost estimations.

There were instances where we may lack control of unobserved confounders in explaining the variance of cost. At the beginning of these studies, we did not have any information about the severity of the comorbid conditions in the patients or whether the disease was under control during that period. For example, for people with hypertension or diabetes, was their blood pressure or blood sugar under control or not during the time of COVID19 infection? We did not include this information in the data. Medicines in regular use by the patients were also not included in the analysis. In addition, there was no observation about the duration of the comorbidities. Another confounder was smoking and alcohol use. We could not analyze whether the patients had habits such as smoking and alcohol or the level of these habits. The patients’ socioeconomic status was another factor that would affect the level of their health status during the illness. In this regard, we did not perform an analysis due to the characteristics of patient registration. Therefore, it will have to be taken into account that we cannot include these variables in the estimates of the determinants of health care costs.

During the first months of the COVID-19 pandemic, physicians tended to hospitalize the patients for close monitoring regardless of severity. However, that practice changed over time, and only patients with higher disease severity, lower oxygen saturation, comorbid conditions, and evidence on chest CT were hospitalized. In fact, in September 2020, the WHO recommended outpatient monitoring of patients with mild to moderate disease [29]. Therefore, this change in treatment approach may have resulted in a lower number of inpatients in the months following the first peak and higher hospital costs among hospitalized patients.

This study also only estimated the direct cost to the health care system and not the substantial mental health burden on society and, in particular, health care professionals during the pandemic. This is also worth investigating further.

Another issue regarding the cost of the disease was the stigmatization and alienation of health care professionals by the general population due to their potential exposure to COVID-19. The economic impact of the pandemic on hospitals, which were forced to restrict access to health care services for patients who did not have COVID-19 but needed care due to other diseases, seems to be another issue that requires further investigation.

## Conclusion

COVID-19 in the first year resulted in a direct annual medical cost burden of PPP$ 2.1 billion in Turkey. It is not easy to estimate the economic cost of the COVID-19 pandemic. The true total cost of the pandemic is undoubtedly significantly higher than the direct medical costs. However, we estimate that the cost of direct health care services for COVID patients was around 2.0% of the overall health care spending.

Of course, it is not possible to predict the source and timing of a new pandemic. The ability to estimate the changes caused by pandemics in the use of health care resources and their potential impact on medical costs will be important for hospitals to develop strategies to manage pandemics.

## Supplementary Information

**Additional file 1.**

## Data Availability

The datasets generated and/or analyzed during the current study are not publicly available due to Turkish Personal Data Protection Law no 6698 but are available from the corresponding author on reasonable request.

## Abbreviations

ALT: Alanine aminotransferase
AST: Aspartate aminotransferase
CI: Confidence Intervals
COPD: Chronic obstructive pulmonary disease
COVID-19: Coronavirus disease 2019
CRD: Chronic renal disease
CRP: C-reactive protein
CT: Computed tomography
GDP: Gross domestic product
GLM: generalized linear model
HCRU: Healthcare resource use
HF: Heart failure
HIN: Health Implementation Notification
ICD-10: International statistical classification of diseases and related health problems 10th revision
ICU: Intensive care unit
IHD: Ischemic heart disease
LDH: Lactate dehydrogenase
LOS: Length of stay
MoH: The Republic of Turkey Ministry of Health
OECD: Organisation for Economic Co-operation and Development
OR: Odds ratio
PaO2/FiO2: ratio of arterial partial pressure of oxygen to fraction of inspired oxygen
PCR: real time reverse transcriptase polymerase chain reaction
PPP: Purchasing power parity
SARS-CoV-2: Severe acute respiratory syndrome coronavirus 2
SpO2: Oxygen saturation
SSI: The Republic of Turkey, Ministry of Family, Labour and Social Services Social Security Institution
TRY: Turkish Lira
WBC: White blood cells
WHO: World Health Organization
