# Supplementary material for: COVID-19 healthcare cost and length of hospital stay in Turkey: retrospective analysis from the first peak of the pandemic
Source: Health Econ Rev. 2021 Oct 8;11:39. doi: 10.1186/s13561-021-00338-8 (PMC8500269; doi:10.1186/s13561-021-00338-8)
Supplement: Supplementary file 1 — Additional file 1. [file 13561_2021_338_MOESM1_ESM.docx]

**Figure F1 Heteroscedasticity for the model**

**
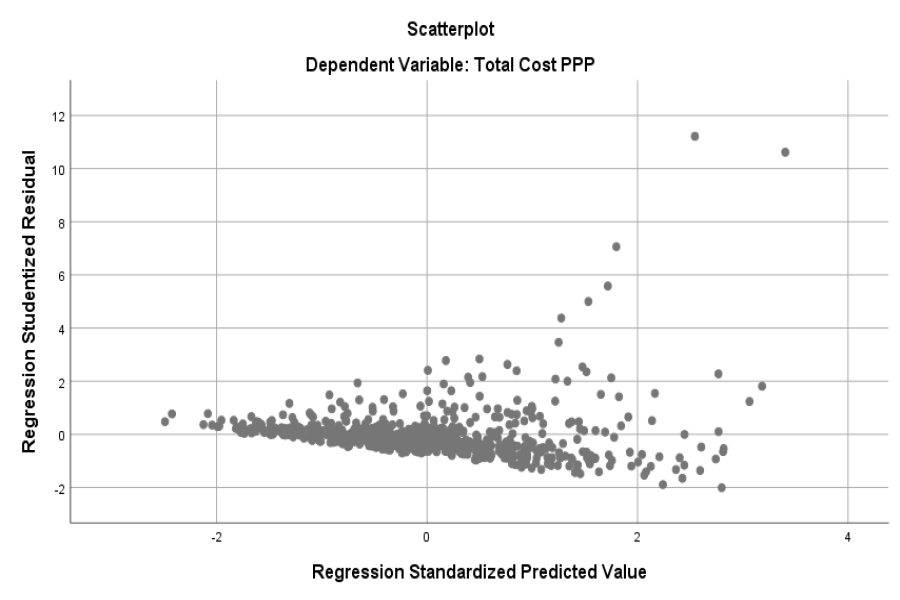
**

**
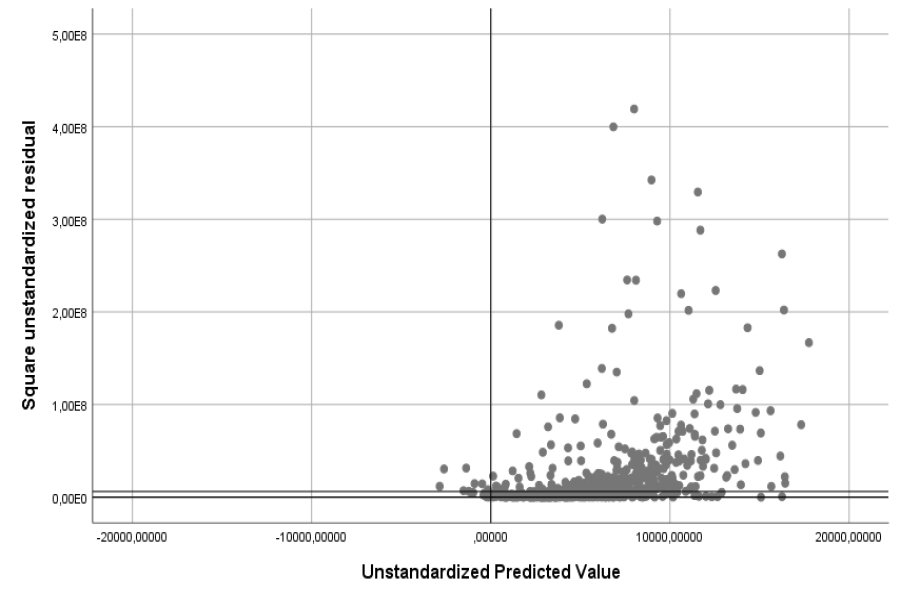
**

**Table S1 Baseline patient demographics, symptoms, findings, and fatality by gender and disease severity**

|  | **Female, n (%)** | | | | | **Male, n (%)** | | | | | |
| --- | --- | --- | --- | --- | --- | --- | --- | --- | --- | --- | --- |
|  | **Mild** | **Moderate** | **Severe** | **Total** | **p-value** | **Mild** | **Moderate** | **Severe** | **Total** | **p-value** |  |
|  | **n=117** | **n=197** | **n=160** | **n=474** |  | **n=124** | **n=238** | **n=220** | **n=582** |  |  |
| **Clinical features** | | | | | | | | | | | |
| Cough | 38 (32.5) | 88 (44.7) | 53 (33.1) | 179 (37.8) | 0.032 | 34 (27.4) | 106 (44.5) | 69 (31.4) | 209 (35.9) | 0.001 |  |
| Fever | 27 (23.1) | 50 (25.4) | 51 (31.9) | 128 (27.0) | 0.212 | 45 (36.3) | 91 (38.2) | 75 (34.1) | 211 (36.3) | 0.654 |  |
| Dyspnea | 0 (0.0) | 79 (40.1) | 61 (38.1) | 140 (29.5) | 0.000 | 0 (0.0) | 89 (37.4) | 93 (42.3) | 182 (31.3) | 0.000 |  |
| Malaise | 17 (14.5) | 44 (22.3) | 36 (22.5) | 97 (20.5) | 0.169 | 20 (16.1) | 37 (15.5) | 42 (19.1) | 99 (17.0) | 0.169 |  |
| Myalgia | 19 (16.2) | 19 (9.6) | 8 (5.0) | 46 (9.7) | 0.002 | 6 (4.8) | 16 (6.7) | 6 (2.7) | 28 (4.8) | 0.234 |  |
| Nausea or vomiting | 8 (6.8) | 17 (8.6) | 12 (7.5) | 37 (7.8) | 0.885 | 3 (2.4) | 18 (7.6) | 7 (3.2) | 28 (4.8) | 0.873 |  |
| Diarrhea | 7 (6.0) | 14 (7.1) | 10 (6.3) | 31 (6.5) | 0.912 | 8 (6.5) | 11 (4.6) | 15 (6.8) | 34 (5.8) | 0.743 |  |
| Headache | 6 (5.1) | 11 (5.6) | 16 (10.0) | 33 (7.0) | 0.096 | 9 (7.3) | 13 (5.5) | 2 (0.9) | 24 (4.1) | 0.003 |  |
| Sputum | 4 (3.4) | 9 (4.6) | 3 (1.9) | 16 (3.4) | 0.377 | 2 (1.6) | 7 (2.9) | 13 (5.9) | 22 (3.8) | 0.120 |  |
| Sore throat | 6 (5.1) | 11 (5.6) | 5 (3.1) | 22 (4.6) | 0.389 | 7 (5.6) | 4 (1.7) | 4 (1.8) | 15 (2.6) | 0.077 |  |
| Chills or rigors | 3 (2.6) | 6 (3.0) | 7 (4.4) | 16 (3.4) | 0.726 | 4 (3.2) | 9 (3.8) | 5 (2.3) | 18 (3.1) | 0.676 |  |
| Chest pain or back pain | 4 (3.4) | 13 (6.6) | 4 (2.5) | 21 (4.4) | 0.339 | 1 (0.8) | 4 (1.7) | 4 (1.8) | 9 (1.5) | 0.331 |  |
| Anorexia | 2 (1.7) | 7 (3.6) | 4 (2.5) | 13 (2.7) | 0.456 | 2 (1.6) | 4 (1.7) | 6 (2.7) | 12 (2.1) | 0.282 |  |
| Abdominal pain | 4 (3.4) | 2 (1.0) | 3 (1.9) | 9 (1.9) | 0.280 | 1 (0.8) | 3 (1.3) | 2 (0.9) | 6 (1.0) | 0.611 |  |
| Loss of smell or taste | 2 (1.7) | 1 (0.5) | 3 (1.9) | 6 (1.3) | 0.514 | 1 (0.8) | 3 (1.3) | 2 (0.9) | 6 (1.0) | 0.611 |  |
| Rhinorrhoea/Stuffiness | 2 (1.7) | 2 (1.0) | 0 (0.0) | 4 (0.8) | 0.111 | 1 (0.8) | 1 (0.4) | 1 (0.5) | 3 (0.5) | 0.490 |  |
| Others | 7 (6.0) | 6 (3.0) | 13 (8.1) | 26 (5.5) | 0.203 | 3 (2.4) | 9 (3.8) | 13 (5.9) | 25 (4.3) | 0.070 |  |
| **Hematologic biomarkers** | | | | | | | | | | | |
| Lymphopenia | 21 (17.9) | 33 (16.8) | 59 (36.9) | 113 (23.8) | 0.000 | 29 (23.4) | 62 (26.1) | 86 (39.1) | 177 (30.4) | 0.002 |  |
| Thrombocytopenia | 17 (14.5) | 30 (15.2) | 35 (21.9) | 82 (17.3) | 0.094 | 32 (25.8) | 44 (18.5) | 74 (33.6) | 150 (25.8) | 0.001 |  |
| Neutrophilia | 13 (11.1) | 22 (11.2) | 31 (19.4) | 66 (13.9) | 0.036 | 18 (14.5) | 40 (16.8) | 53 (24.1) | 111 (19.1) | 0.020 |  |
| **Coagulation biomarkers** | | | | | | | | | | |  |
| Elevated D-dimer | 70 (59.8) | 125 (63.5) | 117 (73.1) | 312 (65.8) | 0.047 | 56 (45.2) | 143 (60.1) | 152 (69.1) | 351 (60.3) | 0.000 |  |
| Elevated fibrinogen | 86 (73.5) | 149 (75.6) | 130 (81.3) | 365 (77.0) | 0.447 | 101 (81.5) | 182 (76.5) | 189 (85.9) | 472 (81.1) | 0.739 |  |
| **Inflammatory biomarkers** | | | | | | | | | | | |
| Elevated C-reactive protein |  | | | | | | | | | |  |
| *3.1-41.8 mg/L* | 51 (43.6) | 113 (57.4) | 70 (43.8) | 234 (49.4) | 0.000 | 59 (47.6) | 102 (42.9) | 65 (29.5) | 226 (38.8) | 0.000 |  |
| *>41.8 mg/L* | 34 (29.1) | 57 (28.9) | 72 (45.0) | 163 (34.4) |  | 45 (36.3) | 119 (50.0) | 147 (66.8) | 311 (53.4) |  |  |
| Elevated serum ferritin | 10 (8.5) | 23 (11.7) | 35 (21.9) | 68 (14.3) | 0.002 | 35 (28.2) | 65 (27.3) | 107 (48.6) | 207 (35.6) | 0.000 |  |
| **Biochemical biomarkers** | | | | | | | | | | | |
| Elevated LDH | 17 (14.5) | 53 (26.9) | 71 (44.4) | 141 (29.7) | 0.000 | 34 (27.4) | 90 (37.8) | 113 (51.4) | 237 (40.7) | 0.000 |  |
| Elevated liver enzymes | 15 (12.8) | 34 (17.3) | 45 (28.1) | 94 (19.8) | 0.001 | 36 (29.0) | 69 (29.0) | 85 (38.6) | 190 (32.6) | 0.056 |  |
| Elevated serum blood urea | 86 (73.5) | 149 (75.6) | 124 (77.5) | 359 (75.7) | 0.739 | 115 (92.7) | 199 (83.6) | 207 (94.1) | 521 (89.5) | 0.001 |  |
| Elevated serum blood creatinine | 10 (8.5) | 27 (13.7) | 33 (20.6) | 70 (14.8) | 0.005 | 24 (19.4) | 53 (22.3) | 57 (25.9) | 134 (23.0) | 0.151 |  |
| **Chest CT results** | | | | | | | | | | | |
| Positive | 0 (0.0) | 181 (91.9) | 82 (51.3) | 263 (55.5) | 0.000 | 0 (0.0) | 211 (88.7) | 132 (60.0) | 343 (58.9) | 0.000 |  |
| **PCR results** | | | | | | | | | | | |
| Positive | 57 (48.7) | 98 (49.7) | 83 (51.9) | 238 (50.2) | 0.766 | 75 (60.5) | 105 (44.1) | 119 (54.1) | 299 (51.4) | 0.007 |  |
| **Comorbidities** | | | | | | | | | | | |
| Comorbidity presence (any) | 54 (46.2) | 102 (51.8) | 85 (53.1) | 241 (50.8) | 0.484 | 46 (37.1) | 99 (41.6) | 107 (48.6) | 252 (43.3) | 0.094 |  |
| Hypertension | 20 (17.1) | 57 (28.9) | 49 (30.6) | 126 (26.6) | 0.022 | 24 (19.4) | 49 (20.6) | 53 (24.1) | 126 (21.6) | 0.524 |  |
| Diabetes | 14 (12.0) | 34 (17.3) | 32 (20.0) | 80 (16.9) | 0.090 | 11 (8.9) | 25 (10.5) | 28 (12.7) | 64 (11.0) | 0.290 |  |
| Asthma/COPD | 5 (4.3) | 23 (11.7) | 21 (13.1) | 49 (10.3) | 0.022 | 8 (6.5) | 21 (8.8) | 18 (8.2) | 47 (8.1) | 0.687 |  |
| Ischemic heart disease | 5 (4.3) | 13 (6.6) | 12 (7.5) | 30 (6.3) | 0.322 | 10 (8.1) | 16 (6.7) | 30 (13.6) | 56 (9.6) | 0.049 |  |
| Cancer | 6 (5.1) | 12 (6.1) | 12 (7.5) | 30 (6.3) | 0.458 | 9 (7.3) | 12 (5.0) | 29 (13.2) | 50 (8.6) | 0.023 |  |
| Chronic renal disease | 4 (3.4) | 11 (5.6) | 6 (3.8) | 21 (4.4) | 0.549 | 4 (3.2) | 12 (5.0) | 10 (4.5) | 26 (4.5) | 0.377 |  |
| Heart failure | 3 (2.6) | 10 (5.1) | 10 (6.3) | 23 (4.9) | 0.107 | 0 (0.0) | 11 (4.6) | 10 (4.5) | 21 (3.6) | 0.026 |  |
| **Inpatient settings** | | | | | | | | | | | |
| Ward only | 111 (94.9) | 178 (90.4) | 127 (79.4) | 416 (87.8) | 0.000 | 116 (93.5) | 210 (88.2) | 151 (68.6) | 477 (82.0) | 0.000 |  |
| Ward &ICU | 6 (5.1) | 19 (9.6) | 33 (20.6) | 58 (12.2) |  | 8 (6.5) | 28 (11.8) | 69 (31.4) | 105 (18.0) |  |  |
| **Fatality** | | | | | | | | | | | |
| Fatality (in hospital) | 3 (2.6) | 8 (4.1) | 23 (14.4) | 34 (7.2) | 0.000 | 3 (2.4) | 18 (7.6) | 45 (20.5) | 66 (11.3) | 0.000 |  |

LDH: Lactate dehydrogenase; CT: Computed tomography; PCR: polymerase chain reaction; SpO2: oxygen saturation

**Table S2 - Comparison of Length of Stay and Costs by Patient Characteristics (univariate)**

|  | **Length of stay (days)** | | | **Total costs (PPP$)** | | |
| --- | --- | --- | --- | --- | --- | --- |
|  | **Mean±SD** | **Mean Difference (95% CI)** | **p-value** | **Mean±SD** | **Mean Difference (95% CI)** | **p-value** |
| **Sex** | | | | | | |
| Male (n=582) | 9.6±7.4 | 1.1 (0.3-1.9) | 0.010 | 6,229.3±8,463.5 | 1,495.7 (623.3-2,368.1) | 0.001 |
| Female (n=474) | 8.5±6.1 |  |  | 4,733.6±5,945.8 |  |  |
| **Hematologic biomarkers** | | | | | | |
| Lymphopenia |  |  |  |  |  |  |
| Yes (n=290) | 10.9±9.8 | 2.6 (1.4-3.8) | 0.000 | 8,416.5±11,917.3 | 3,940.8 (2,529.4—5,352.3) | 0.000 |
| No (n=766) | 8.4±5.2 |  |  | 4,475.7±4,370.5 |  |  |
| Thrombocytopenia |  |  |  |  |  |  |
| Yes (n=232) | 9.9±6.5 | 1.1 (0.1-2.1) | 0.033 | 6,241.5±6,307.5 | 876.1 (-231.1-1,965.2) | 0.115 |
| No (n=824) | 8.8±6.9 |  |  | 5,365.5±7,762.8 |  |  |
| Neutrophilia |  |  |  |  |  |  |
| Yes (n=177) | 10.7±10.0 | 1.9 (0.4-3.4) | 0.016 | 9,434.5±13,648.4 | 4,657.1 (2,604.9-6,709.3) | 0.000 |
| No (n=879) | 8.8±6.0 |  |  | 4,777.3±5,111.9 |  |  |
| **Coagulation biomarkers** | | | | | | |
| Elevated D-dimer |  |  |  |  |  |  |
| Yes (n=663) | 9.7±7.2 | 1.6 (0.8-2.4) | 0.000 | 6,514.3±8,661.0 | 2,602.0 (1,810.6-3,393.4) | 0.000 |
| No (n=391) | 8.1±6.2 |  |  | 3911,8±4400,5 |  |  |
| Elevated fibrinogen |  |  |  |  |  |  |
| Yes (n=837) | 9.5±7.3 | 1.4 (0.2-2.7) | 0.022 | 6,086.4±8,047.8 | 1,475.2 (555.6—2,394.7) | 0.002 |
| No (n=146) | 8.1±5.1 |  |  | 4,611.2±4,539.2 |  |  |
| **Inflammatory biomarkers** | | | | | | |
| Elevated C-reactive protein |  |  |  |  |  |  |
| Normal <3 (n=122) | 6.7±3.9 |  |  |  |  |  |
| Elevated >3 <41.8 (n=460) | 8.4±5.7 | 1.7 (0.6-2.8)* | 0.000 | 4,323.4±4,726.6 | 1,353.5 (594.4-2,112.5)* | 0.000 |
| Elevated >41,8 (n=474) | 10.4±8.1 | 3.8 (2.5-5.0)* | 0.000 | 7,422.2±9,725.9 | 4,452.3 (3,252.3-5,652.2)* | 0.000 |
| Elevated serum ferritin |  |  |  |  |  |  |
| Yes (n=275) | 11.8±9.8 | 3.5 (2.3-4.7) | 0.000 | 8,985.3±11,593.0 | 4,466.6 (3,046.7-5,886.5) | 0.000 |
| No (n=731) | 8.3±5.2 |  |  | 4,518.7±4,838.3 |  |  |
| **Biochemical biomarkers** | | | | | | |
| Elevated LDH |  |  |  |  |  |  |
| Yes (n=378) | 10.6±7.8 | 2.2 (1.3-3.2) | 0.000 | 7,789.7±10,353.4 | 3,447.8 (2,339.0-4,556.7) | 0.000 |
| No (n=667) | 8.3±6.2 |  |  | 4,341.9±4,820.3 |  |  |
| Elevated liver enzymes |  |  |  |  |  |  |
| Yes (n=284) | 10.6±8.5 | 2.1 (1.0-3.2) | 0.000 | 7,503.4±11,018.1 | 2,661.2 (1,318.0-4,004.3) | 0.000 |
| No (n=772) | 8.5±6.0 |  |  | 4,842.2±5,473.4 |  |  |
| Elevated serum blood urea |  |  |  |  |  |  |
| Yes (n=880) | 9.4±7.2 | 1.5 (0.7-2.3) | 0.000 | 5,897.4±8,008.0 | 1,980.3 (1,244.3-2,716.4) | 0.000 |
| No (n=173) | 7.8±4.2 |  |  | 3,917.0±3,423.0 |  |  |
| Elevated serum blood creatinine |  |  |  |  |  |  |
| Yes (n=204) | 10.1±8.2 | 1.2 (0.0-2.4) | 0.048 | 7,546.9±9,998.2 | 2,449.5 (999.2—3,899.7) | 0.001 |
| No (n=849) | 8.9±6.5 |  |  | 5,097.5±6,656.2 |  |  |
| **Chest CT** | | | | | | |
| Positive (n=606) | 9.6±7.4 | 1.9 (0.7-3.2) | 0.003 | 6,071.6±8,193.3 | 2,075.9 (1,073.8-3,078.0) | 0.000 |
| Negative (n=156) | 7.7±6.5 |  |  | 3,995.7±4,822.8 |  |  |
| **PCR (2 samples)** | | | | | | |
| Positive (n=537) | 9.9±7.6 | 1.7 (0.9-2.5) | 0.000 | 6,406.0±8,880.2 | 1,641.6 (736.6-2,546.6) | 0.000 |
| Negative (n=488) | 8.2±6.0 |  |  | 4,764.4±5,666.7 |  |  |
| **O2 Saturation** | | | | | | |
| <94% (n=368) | 10.9±8.5 | 2.5 (1.5-3.5) | 0.000 | 7,587.5±9,386.2 | 2,869.4 (1,795.4-3,943.4) | 0.000 |
| ≥%94 (n=627) | 8.4±5.7 |  |  | 4,718.1±6,111.3 |  |  |
| **Comorbidity presence** | | | | | | |
| Yes (n=493) | 9.9±8.5 | 1.5 (0.6-2.3) | 0.001 | 6,618.5±8,555.7 | 1,989.3 (1,073.8-2,904.8) | 0.000 |
| No (n=563) | 8.4±4.9 |  |  | 4,629.2±6,238.1 |  |  |
| Hypertension |  |  |  |  |  |  |
| Yes (n=252) | 9.5±6.5 | 0.5 (-0.4-1.5) | 0.270 | 6,390.6±6,651.4 | 1,093.7 (36.6-2,150.8) | 0.043 |
| No (n=804) | 9.0±7.0 |  |  | 5,296.9±7,698.4 |  |  |
| Diabetes |  |  |  |  |  |  |
| Yes (n=144) | 10.3±8.7 | 1.4 (0.2-2.6) | 0.025 | 6,966.7±9,953.3 | 1,631.2 (-68.9-3,331.2) | 0.060 |
| No (n=912) | 8.9±6.5 |  |  | 5,335.5±6,983.3 |  |  |
| Asthma/COPD |  |  |  |  |  |  |
| Yes (n=96) | 10.3±10.4 | 1.3 (-0.8-3.5) | 0,230 | 6,744.8±8,238.8 | 1,305.5 (-263.0-2,874.0) | 0,238 |
| No (n=960) | 9.0±6.4 |  |  | 5,439.3±7,386.7 |  |  |
| Ischemic heart disease |  |  |  |  |  |  |
| Yes (n=86) | 10.0±5.7 | 1.1 (-0.4-2.6) | 0.168 | 7,053.5±6,215.7 | 1,628.2 (-19.6-3,276.0) | 0.053 |
| No (n=970) | 9.0±6.9 |  |  | 5,425.3±7,563.3 |  |  |
| Cancer |  |  |  |  |  |  |
| Yes (n=80) | 11.4±11.0 | 2.5 (-0.1-4.9) | 0.053 | 8,699.6±12,856.7 | 3,399.2 (507.5-6,290.9) | 0.022 |
| No (n=976) | 8.9±6.4 |  |  | 5,300.4±6,794.6 |  |  |
| Chronic renal disease |  |  |  |  |  |  |
| Yes (n=47) | 10.4±6.9 | 1.4 (-0.6-3.4) | 0.169 | 7,895.0±7,144.1 | 2,445.9 (261.5-4,630.2) | 0.028 |
| No (n=1009) | 9.0±6.8 |  |  | 5,449.1±7,474.0 |  |  |
| Heart failure |  |  |  |  |  |  |
| Yes (n=44) | 10.9±8.0 | 1.9 (-0.2-4.0) | 0.071 | 8,687.9±9,488.5 | 3,266.0 (348.1-6,183.9) | 0.029 |
| No (n=1012) | 9.0±6.8 |  |  | 5,421.9±7,349.1 |  |  |
| **O2 Therapy** | | | | | | |
| Yes (n=613) | 10.6±8.0 | 3.7 (3.0-4.4) | 0.000 | 6,889.5±9,093.8 | 3,174.2 (2,379.0-3,969.4) | 0.000 |
| No (n=443) | 6.9±3.8 |  |  | 3,715.3±3,598.0 |  |  |
| **Fatality** | | | | | | |
| Yes (n=100) | 13.7±11.5 | 5.1 (3.8-6.5) | 0.000 | 15,327.6±17,454.2 | 10,791.5 (7,317.5-14,265.6) | 0.000 |
| No (n=956) | 8.6±6.0 |  |  | 4,536.0±4,368.0 |  |  |

**Table S3 Associations with characteristics and total hospital costs based on generalized linear model**

| **Characteristics** | **Exp[β] (95% CI)** | **p-value** |
| --- | --- | --- |
| Age (>65) x Sex (Male) | 4.573 (1.109-18.855) | 0.035 |
| Age (>65) x Sex (Female) | 5.817 (1.572-21.533) | 0.008 |
| Age (<65) x Sex (Male) | 1.968 (0.977-3.965) | 0.058 |
| **Disease severity** |  |  |
| Moderate | 0.616 (0.234-1.619) | 0.326 |
| Severe | 1.038 (0.417-2.580) | 0.937 |
| **Hematologic biomarkers** |  |  |
| Lymphopenia | 1.385 (0.489-3.924) | 0.540 |
| Thrombocytopenia | 1.171 (0.674-2.033) | 0.575 |
| Neutrophilia | 1.890 (1.116-3.199) | 0.018 |
| **Coagulation biomarkers** |  |  |
| Elevated D-dimer | 0.677 (0.381-1.202) | 0.183 |
| Elevated fibrinogen | 2.037 (0.883-4.698) | 0.095 |
| **Inflammatory biomarkers** |  |  |
| Elevated C-reactive protein |  |  |
| 3.1-41.8 mg/L | 1.455 (0.823-2.572) | 0.197 |
| >41.8 mg/L | 1.486 (0.780-2.830) | 0.228 |
| Elevated serum ferritin | 0.903 (0.537-1.520) | 0.702 |
| **Biochemical biomarkers** |  |  |
| Elevated LDH | 1.656 (1.120-2.449) | 0.011 |
| Elevated liver enzymes | 1.758 (0.965-3.205) | 0.065 |
| Elevated serum blood urea | 0.905 (0.520-1.574) | 0.722 |
| Elevated serum blood creatinine | 0.988 (0.424-2.302) | 0.977 |
| **Serology** |  |  |
| PCR Positive | 4.013 (2.035-7.916) | 0.000 |
| **Respiratory status** |  |  |
| Chest CT Positive | 1.417 (0.436-4.601) | 0.562 |
| O2 theraphy | 2.027 (1.301-3.160) | 0.002 |
| **Comorbidities** |  |  |
| Comorbidity any | 1.497 (0.753-2.974) | 0.250 |
| Hypertension | 3.284 (1.045-10.317) | 0.042 |
| Diabetes | 0.570 (0.196-1.656) | 0.301 |
| Asthma/COPD | 1.485 (0.700-3.147) | 0.303 |
| Ischemic heart disease | 0.284 (0.082-0.983) | 0.047 |
| Cancer | 1.667 (0.532-5.223) | 0.380 |
| Chronic renal disease | 0.167 (0.038-0.735) | 0.018 |
| Heart failure | 0.315 (0.094-1.056) | 0.061 |

**Table S4 The age (>65 year) interactions with covariates, and total hospital costs based on generalized linear model**

| **Characteristics** | **Exp[β] (95% CI)** | **Standard error** | **p-value** |
| --- | --- | --- | --- |
| **Age >65** | | | |
| Sex (male) | 6.063 (2.239-16.416) | 0.508 | 0.000 |
| **Disease severity** |  |  |  |
| Moderate | 0.889 (0.356-2.223) | 0.468 | 0.802 |
| Severe | 1.314 (0.435-3.972) | 0.564 | 0.628 |
| **Hematologic biomarkers** |  |  |  |
| Lymphopenia | 1.001 (0.446-2.250) | 0.413 | 0.997 |
| Thrombocytopenia | 0.925 (0.528-1.620) | 0.286 | 0.784 |
| Neutrophilia | 1.403 (0.517-3.809) | 0.510 | 0.506 |
| **Coagulation biomarkers** |  |  |  |
| Elevated D-dimer | 0.864 (0.511-1.463) | 0.268 | 0.587 |
| Elevated fibrinogen | 1.242 (0.821-1.877) | 0.211 | 0.304 |
| **Inflammatory biomarkers** |  |  |  |
| Elevated C-reactive protein |  |  |  |
| 3.1-41.8 mg/L | 1.070 (0.664-1.725) | 0.244 | 0.780 |
| >41.8 mg/L | 1.078 (0.491-2.368) | 0.401 | 0.851 |
| Elevated serum ferritin | 1.666 (0.862-3.220) | 0.336 | 0.129 |
| **Biochemical biomarkers** |  |  |  |
| Elevated LDH | 1.116 (0.588-2.117) | 0.327 | 0.738 |
| Elevated liver enzymes | 1.385 (0.487-3.936) | 0.533 | 0.541 |
| Elevated serum blood urea | 0.754 (0.400-1.422) | 0.324 | 0.384 |
| Elevated serum blood creatinine | 1.112 (0.717-1.724) | 0.224 | 0.635 |
| **Serology** |  |  |  |
| PCR Positive | 1.225 (0.707-2.124) | 0.281 | 0.469 |
| **Respiratory status** |  |  |  |
| Chest CT Positive | 1.805 (0.692-4.705) | 0.489 | 0.227 |
| O2 theraphy | 1.603 (0.943-2.723) | 0.271 | 0.081 |
| **Comorbidities** |  |  |  |
| Comorbidity any | 0.921 (0.493-1.719) | 0.319 | 0.795 |
| Hypertension | 1.498 (0.854-2.628) | 0.287 | 0.158 |
| Diabetes | 0.659 (0.363-1.194) | 0.304 | 0.169 |
| Asthma/COPD | 1.685 (0.898-3.162) | 0.321 | 0.104 |
| Ischemic heart disease | 1.377 (0.776-2.443) | 0.293 | 0.274 |
| Cancer | 0.529 (0.177-1.584) | 0.560 | 0.255 |
| Chronic renal disease | 0.621 (0.311-1.240) | 0.353 | 0.177 |
| Heart failure | 1.542 (0.461-5.155) | 0.616 | 0.482 |
